# Data on the use of traditional and complementary medicine, WHO Region of the Americas

**DOI:** 10.2471/BLT.25.293411

**Published:** 2025-09-22

**Authors:** Natalia Houghton, Herfina Nababan, Ernesto Bascolo

**Affiliations:** a Department of Health Systems and Services, Pan American Health Organization, 525 23rd Street NW, Washington DC, 20037, United States of America.; b Department of Global Health, University of Bremen, Bremen, Germany.

## Abstract

An understanding of the population-level data available on the use of traditional, complementary and integrative medicine is critical for reducing unmet health needs and improving health outcomes. Although research has shown that the existence of unmet health-care needs among people receiving conventional health care can drive the use of traditional medicine, the motivations for its use are complex and often related to cultural traditions, personal preferences and perceptions of effectiveness. At present, only limited population-based data are available on who uses traditional medicine, the reasons for its use, the context in which it is used (even when traditional medicine is a primary choice) and the outcomes of treatment. To address this data gap, we identified and analysed population-based surveys that reported data on traditional medicine use for perceived health problems in the World Health Organization’s Region of the Americas. Based on the findings, we discuss how best to analyse available population-based survey data and how survey questions on traditional medicine can be refined to enhance their usefulness. Strengthened data collection on traditional medicine can inform health-care policy on the integration of traditional practices into the health system, aid efforts to educate both health-care providers and the public on traditional medicine, support culturally responsive and people-centred care, and foster the ethical and effective integration of traditional medicine into health systems.

## Résumé

Il est essentiel de bien comprendre les données disponibles au niveau des populations sur l’utilisation de la médecine traditionnelle, complémentaire et intégrative afin de réduire les besoins non satisfaits en matière de santé et d’améliorer les résultats sanitaires. Bien que des recherches aient mis en évidence que l’existence de besoins de santé non satisfaits chez les personnes bénéficiant de soins de santé conventionnels est de nature à favoriser le recours à la médecine traditionnelle, les motivations de son utilisation sont complexes et souvent liées à des traditions culturelles, des préférences personnelles et aux perceptions de son efficacité. À l’heure actuelle, seules des données démographiques limitées sont disponibles sur les utilisateurs de la médecine traditionnelle, les raisons de son utilisation, le contexte dans lequel elle est utilisée (même lorsqu’il s’agit du choix de première intention) et les résultats du traitement. Pour combler cette lacune, nous avons identifié et analysé des enquêtes démographiques qui fournissaient des données sur l’utilisation de la médecine traditionnelle pour traiter des problèmes de santé perçus au sein de la région des Amériques de l’Organisation mondiale de la Santé. Sur la base des résultats, nous discutons de la meilleure façon d’analyser les données disponibles issues d’enquêtes démographiques et de la manière d’affiner les questions relatives à la médecine traditionnelle afin d’améliorer leur utilité. Le renforcement de la collecte de données sur la médecine traditionnelle permet d’éclairer les politiques de santé en matière d’intégration des pratiques traditionnelles, de soutenir les efforts visant à sensibiliser les prestataires de soins de santé et le public à la médecine traditionnelle, de favoriser des soins centrés sur les personnes et adaptés aux différentes cultures et d’encourager l’intégration éthique et efficace de la médecine traditionnelle dans les systèmes de santé.

## Resumen

La comprensión de los datos poblacionales disponibles sobre el uso de la medicina tradicional, complementaria e integrativa es esencial para reducir las necesidades de salud no cubiertas y mejorar los resultados sanitarios. Aunque las investigaciones han demostrado que la existencia de necesidades no satisfechas en la atención sanitaria convencional puede impulsar el uso de la medicina tradicional, las motivaciones de su utilización son complejas y a menudo están relacionadas con tradiciones culturales, preferencias personales y percepciones de eficacia. En la actualidad, solo existen datos poblacionales limitados que describen quién utiliza la medicina tradicional, los motivos de su uso, el contexto en que se emplea (incluso cuando se elige como opción principal) y los resultados del tratamiento. Para abordar esta carencia de datos, se identificaron y analizaron encuestas poblacionales que informaban sobre el uso de la medicina tradicional para problemas de salud percibidos en la Región de las Américas de la Organización Mundial de la Salud. A partir de los hallazgos, se discute la mejor manera de analizar los datos de encuestas poblacionales disponibles y cómo pueden perfeccionarse las preguntas sobre medicina tradicional para aumentar su utilidad. El fortalecimiento de la recopilación de datos sobre medicina tradicional puede orientar las políticas sanitarias hacia la integración de las prácticas tradicionales en el sistema de salud, apoyar las iniciativas de formación tanto de los profesionales sanitarios como del público en general, promover una atención culturalmente pertinente y centrada en las personas, y favorecer la integración ética y eficaz de la medicina tradicional en los sistemas sanitarios.

## ملخص

يُعد فهم البيانات المتاحة على مستوى السكان حول استخدام الطب التقليدي، والطب التكميلي، والطب التكاملي، أمراً بالغ الأهمية لتقليل الاحتياجات الصحية غير المستوفاة وتحسين النتائج الصحية. ورغم أن الأبحاث أظهرت أن وجود احتياجات للرعاية الصحية غير مستوفاة لدى المرضى الذين يتلقون الرعاية الصحية التقليدية، قد يدفعهم إلى استخدام الطب التقليدي، إلا أن دوافع استخدام هذا النوع من الطب معقدة، وغالباً ما ترتبط بالتقاليد الثقافية، والتفضيلات الشخصية، وعناصر إدراك الفعالية. حالياً، تتوفر بيانات محدودة فقط على مستوى السكان حول من يستخدم الطب التقليدي، وأسباب استخدامه، والظروف المحيطة باستخدامه (حتى عندما يكون الطب التقليدي الخيار الأول)، ونتائج العلاج. لمعالجة هذا النقص في البيانات، قمنا بتحديد وتحليل الاستبيانات السكانية التي أنتجت بيانات عن استخدام الطب التقليدي لعلاج المشاكل الصحية المعروفة في منطقة الأمريكتين التابعة لمنظمة الصحة العالمية. استناداً إلى النتائج، نحن نناقش أفضل طرق تحليل بيانات الاستبيان السكاني المتاحة، وكيفية تحسين أسئلة الاستبيان المتعلقة بالطب التقليدي لتعزيز فائدتها. إن تعزيز جمع البيانات حول الطب التقليدي من شأنه أن يساهم في صياغة سياسات الرعاية الصحية لتكامل الممارسات التقليدية في النظام الصحي، ودعم جهود توعية مقدمي الرعاية الصحية والجمهور حول الطب التقليدي، وتعزيز الرعاية الصحية التي تراعي التنوع الثقافي وتضع الإنسان في صلب اهتمامها، وتعزيز التكامل الأخلاقي والفعال للطب التقليدي في أنظمة الرعاية الصحية.

## 摘要

了解传统医学、补充医学和综合医学的人口层面使用数据，对减少未被满足的健康需求和改善健康结果至关重要。尽管研究表明，接受传统医疗护理人群存在未被满足的医疗护理需求可驱动传统医学的使用，但传统医学的使用动机比较复杂，通常与文化传统、个人偏好和对疗效的看法等有关。目前，在传统医学的使用人群、原因和背景（甚至传统医学作为首选的情况）及治疗效果方面仅有一些有限的基于人口的数据。为解决这一数据缺口，我们找到并分析了世卫组织在美洲地区开展的基于人口的相关调查，这些调查报告了使用传统医学来治疗已知健康问题方面的数据。我们根据研究结果讨论了如何以最好的方式来分析基于人口的现有调查数据，以及如何改进传统医学的调查问题，以提高其有用性。加强传统医学数据收集可为医疗护理政策提供将传统实践纳入卫生系统的相关信息，有助于向医疗护理提供者和公众宣传传统医学，为文化响应和以人为本的护理提供支持，并促进以合乎道德和有效的方式将传统医学纳入卫生系统。

## Резюме

Понимание данных популяционного уровня относительно использования традиционной, комплементарной и интегративной медицины крайне важно для сокращения количества неудовлетворенных потребностей в услугах здравоохранения и для улучшения результатов охраны здоровья. Хотя исследования и показали, что наличие неудовлетворенных потребностей в охране здоровья среди лиц, получающих конвенционное лечение, может стать поводом для обращения к традиционной медицине, мотивация ее использования сложна и часто связана с культурными традициями, личными предпочтениями и представлением об эффективности. В настоящее время доступны только ограниченные популяционные данные о том, кто пользуется традиционной медициной, о причинах ее использования, о том контексте, в котором она применяется (даже когда традиционная медицина является первоочередным выбором), и об исходах лечения. Чтобы заполнить этот пробел в данных, авторы выявили и проанализировали популяционные опросы, в которых сообщались данные об использовании традиционной медицины в случаях субъективно воспринимаемых проблем со здоровьем, собранные в Американском регионе ВОЗ. На основе этих результатов авторы обсуждают, как можно наилучшим образом проанализировать имеющиеся данные популяционных опросов и как можно усовершенствовать посвященные традиционной медицине анкетные вопросы с целью повышения их полезности. Получение более надежных данных об использовании традиционной медицины может стать основной для политик в сфере здравоохранения, целью которых будет внедрение традиционных практик в систему здравоохранения, поможет в деле расширения осведомленности как поставщиков услуг здравоохранения, так и общественности в вопросах традиционной медицины, послужит поддержкой для культурно ориентированного, сосредоточенного на пациенте медицинского лечения, а также будет содействовать этичной и эффективной интеграции традиционной медицины в системы здравоохранения.

## Introduction

The term traditional, complementary and integrative medicine (hereafter referred to as traditional medicine) encompasses diverse health practices used worldwide. Traditional medicine itself refers to the knowledge, skills and practices rooted in cultural traditions that aim to maintain health and treat disease. Complementary medicine includes health-care practices that are not part of a country’s conventional health system but are used alongside it; and integrative medicine refers to the combination of traditional and complementary medicine with biomedicine. Traditional medicine plays a vital part in health systems worldwide, either as the mainstay or as a complement. In many countries, it is a component of spiritual and cultural belief systems and a common source of primary care.^1^^,^^2^ Today, people in high-income countries are increasingly embracing traditional medicine to complement standard health care.^3^

The World Health Organization (WHO) recognizes traditional medicine as beneficial and essential for achieving universal health coverage when it is adequately integrated into the health system.^4^ This recognition was reflected in a resolution on traditional medicine at the Sixty-seventh World Health Assembly, which called on Member States to integrate traditional medicine into health systems and services by adapting, adopting and implementing, where appropriate, WHO’s 2014–2023 traditional medicine strategy.^4^^,^^5^ In 2019, 170 Member States reported that traditional medicine was used in their health systems, and many of those States had strengthened national frameworks for traditional medicine, including policies on traditional medicine, offices for traditional medicine and research institutes focused on traditional medicine.^6^

The extent to which health systems accommodate cultural values when providing care remains unclear, which has implications for the use of traditional medicine and for meeting health needs. In addition, consolidated evidence is lacking on the extent to which traditional medicine contributes to efforts to establish universal health coverage. A key problem for policy-makers is the limited availability of robust, population-based data on the use of traditional medicine and its relationship to unmet health needs, particularly given the diverse ways in which traditional medicine has been integrated into health care around the world.

Our study set out to address the gap in population-based data on traditional medicine by analysing how national health surveys can provide evidence on the relationship between traditional medicine and unmet health needs, with reference to recent publications and policy developments. The main questions we examined were: What is the value and what are the limitations of using routine health surveys as a source for policy-relevant data on traditional medicine? We focused on countries in the WHO Region of the Americas because recent, representative population-based data that included references to traditional medicine use were available, and because of the diversity of approaches to integrating traditional medicine in the region, ranging from full legal recognition and insurance coverage to informal or unregulated use.^2^^,^^7^ Our aim was to perform a literature-based policy analysis to guide better data collection and inform policy on traditional medicine, and to promote its ethical and effective integration into health systems, thereby providing a more people-centred and culturally responsive approach to care.^6^

## Evidence on traditional medicine use

Although few assessments of the need for traditional medicine have been conducted at the population level,^1^^,^^8^ several publications have reported on its utilization to meet health-care needs using population-based data. For example, a systematic review identified 40 studies from 14 countries that used 21 national and one cross-national survey to estimate the utilization of traditional medicine by the general population.^9^ Their findings revealed a substantial variation in use: the estimated 12-month prevalence ranged from 24.0% in the 2012 Swiss Health Survey to 71.3% in a 2011 study in the Republic of Korea.^10^^,^^11^ In addition, an analysis of the 2014 European Social Survey covering 21 countries found that an estimated 25.9% of Europeans reported traditional medicine use.^12^ In India, almost half of individuals reported the use of Ayush (i.e. traditional medicine) for the prevention or treatment of illness in the previous year, according to a survey in 2022 and 2023.^13^ Although smaller cross-sectional studies have contributed data on the use of traditional medicine,^8^ no study has explicitly investigated the correlation between its use and unmet health-care needs.

The relationship between conventional health care and traditional medicine is multifaceted. Studies show that people often alternate between conventional health care and traditional healers to meet their health needs,^14^^,^^15^ especially in low- and middle-income countries with pluralistic health systems.^14^ People’s choices, which are often viewed as complementary, are influenced by factors such as personal preference, symptom severity, accessibility, affordability and social influences.^8^

In recognizing the importance of traditional medicine for health and well-being, WHO supports its integration into health systems and has defined integrative medicine as an “interdisciplinary and evidence-based approach to health and well-being by using a combination of biomedical and traditional and/or complementary medical knowledge, skills and practices.”^16^ The integration of different practices along with improved coordination and communication between health-care sectors would be expected to ensure the continuity and effectiveness of care.

As well as a lack of systematic needs assessments for traditional medicine, the WHO Global Traditional Medicine Centre found that limited scientific evidence on the safety and efficacy of many traditional medicine products and practices has also presented a barrier to their integration into health systems.^16^ Moreover, WHO’s 2023 map of systematic reviews on traditional, complementary and integrative medicine revealed substantial deficits in research, particularly for paediatric populations and specific health conditions.^17^

In 2024, researchers outlined additional persistent challenges affecting research, including financial constraints, limited training capacity and methodological issues, such as a lack of standardization within traditional medicine research.^18^ Addressing these challenges will require comprehensive strategies to increase funding, promote research literacy and encourage open science practice. These measures are crucial for building the robust evidence needed to integrate traditional medicine into mainstream health care and to overcome persistent biases and skepticism.^18^

## Unmet health-care needs

Unmet health-care needs drive the use of traditional medicine. An unmet need occurs when an individual cannot access, or benefit from, health services despite having a health concern. These needs may arise from barriers such as distance from a facility, high costs, long waiting times and language or cultural differences, or an experience of discrimination in the health system.^19^

Although unmet needs often prompt the use of traditional medicine, they are not the sole motivation. In many contexts, traditional medicine is the preferred, first-line option because of cultural beliefs, trust or perceived benefits, not only when other options are unavailable.^20^^,^^21^ A recognition of these factors is critical for understanding the role of traditional medicine in meeting health needs and in designing a people-centred health system.

Traditional medicine often serves as the primary or routine source of care, especially where there is a cultural preference, trust in local practitioners, a holistic view of health or a perception that it is safe and has fewer side-effects.^1^^,^^8^^,^^22^^–^^27^ In some settings, for example in China, India and the Republic of Korea, the formal integration of traditional medicine into the health system further supports its mainstream use.^9^^,^^13^^,^^22^^,^^23^^,^^28^

In addition, the use of traditional medicine is frequently a response to barriers in the conventional health system, such as limited access, long waiting times or perceived discrimination, especially in low- and middle-income countries and rural or underserved areas.^3^^,^^8^^,^^23^^,^^24^^,^^29^^,^^30^ In this context, traditional medicine can provide a crucial source of care for people unable to access mainstream services, thereby filling an important gap in health care.

Studies in several countries confirm that individuals with chronic health conditions or unmet health-care needs and those who perceive biomedical care as limited are more likely to turn to traditional medicine. For example, researchers found that adults in Canada with chronic pain and unmet needs had a higher use of traditional medicine.^29^ High traditional medicine uptake was also observed in parts of sub-Saharan Africa where there were few biomedical care providers.^28^ Peer recommendations and family testimony can further influence the choice of care.^23^

Affordability is also a key motivator: in many low- and middle-income countries, traditional medicine may be less costly than conventional care, which encourages its use among low-income groups.^23^^,^^24^ However, findings from Nepal and high-income countries show that higher income and education are also associated with traditional medicine use, which implies there are motivations beyond economic necessity.^8^

Cultural beliefs, privacy concerns, a lack of trust in biomedical care and the holistic appeal of traditional medicine all influence care-seeking behaviour. Traditional approaches may be favoured for their use of natural products, fewer side-effects and responsiveness to emotional, spiritual or sensitive health issues.^8^^,^^23^^,^^27^^,^^29^^,^^31^^,^^32^

The burden of chronic noncommunicable disease has also driven greater traditional medicine use, particularly where therapies such as acupuncture or medicinal plants have recognized benefits; for example, in smoking cessation or symptom management among cancer patients.^33^^,^^34^ In Germany, over half of individuals surveyed in 2024 preferred an approach that combined traditional medicine and conventional care.^33^

Despite its benefits, traditional medicine is not without risks. In a 2023 technical report, WHO emphasized that traditional medicine should be integrated into health care using a science-based approach to ensure its safety, quality and effectiveness.^35^ Rigorous evaluations should be conducted, regulation should be standardized and patients should have financial protection. Understanding the link between unmet health-care needs and the use of traditional medicine could help tailor health systems and make them more effective, equitable and people-centred by drawing on the strengths of both traditional and conventional medicine.^35^

## Population-based surveys

Population-based surveys, such as national health and household surveys, are practical and cost-effective tools for assessing how and when people use traditional medicine. Although specialized national surveys would offer more comprehensive data, they are rarely conducted because of logistical, financial and capacity constraints.^19^ A feasible alternative is to incorporate questions on traditional medicine into existing national survey platforms and programmes such as WHO’s World Health Survey Plus,^36^ which is currently underway.

To explore the potential of population-based surveys to provide data on traditional medicine use, we reviewed nationally representative surveys from 18 countries in the WHO Region of the Americas that included questions on unmet health needs.^7^^,37^ To maximize their representativeness, these surveys were identified through systematic searches of public data repositories, including those of national statistical offices and health ministries.^7^ Inclusion criteria were: (i) national representativeness; (ii) the availability of microdata or comprehensive documentation; (iii) the presence of at least one traditional medicine-related response category; and (iv) a clear description of the sampling method and weighting procedures used. Although efforts were made to include all countries in the region, many lacked recent or sufficiently detailed surveys, or underrepresented Indigenous groups due to sampling and response constraints.

We identified 11 countries that had conducted at least one survey between 2010 and 2023 and whose survey included a traditional medicine-related response category related either to care-seeking behaviour or the reason for forgoing conventional care (Table 1). Use of traditional medicine was typically included as a possible response to questions about health-care seeking or utilization. Respondents were generally asked where they sought or received care for their most recent medical issue, and were often presented with several options to choose from, such as a conventional health-care provider, a traditional medicine provider, self or home care, or not doing anything. Such data can provide useful estimates of, for example, the proportion of the population with a perceived health problem that use traditional medicine compared to conventional care or doing nothing. Moreover, these data can be disaggregated by demographic characteristics or health conditions to better understand who tends to use traditional medicine.

**Table 1:** Typical household survey questions on traditional medicine use, review of population-based surveys reporting traditional medicine use, WHO Region of the Americas, 2010–2023

Survey topic and response options	Country examples of where these response options were presented	Interpretation of responses and additional questions	Comments on response interpretation
**Type of care used for an illness or accident**			
- Received care by ancestral medicine or complementary medicine - Was seen by a folk healer, herbalist or naturopath - Traditional midwife - Healer - Naturopath	Ecuador, Guatemala and Honduras	(i) Traditional medicine met health-care needs; and (ii) there were additional questions on barriers to care but not for traditional medicine users	Traditional medicine may be regarded as a substitute for conventional health care, thereby potentially masking unmet needs
- Attended a complementary medicine clinic (for example, acupuncture or reiki)^a^ - Consulted a traditional doctor - Was seen at a healer's or midwife's house	Barbados, Bolivia (Plurinational State of) and Nicaragua	(i) Traditional medicine met health-care needs; and (ii) there were no additional questions on barriers to care	Traditional medicine may be regarded as a substitute for conventional health care, thereby potentially masking unmet needs
- Consulted an empirical healer, folk healer, herbalist or midwife - Received complementary therapy (for example, acupuncture, flower essences, music therapy or homeopathy)^a^ - Healer - Traditional midwife - Naturopath (not a doctor)	Colombia, El Salvador and the Dominican Republic	(i) Traditional medicine met health-care needs; and (ii) there were additional questions on barriers to care but only for respondents who chose traditional medicine	The use of traditional medicine may be incorrectly assumed to indicate an unmet need for health care, thereby overlooking the possibility it was chosen because of cultural preferences or was being used alongside conventional medicine as a complementary treatment
**Reason for not seeking medical care**			
- Preferred to consult a specialist in complementary medicine (for example, vibrational medicine, biomagnetism, reiki, iridology, Bach flower remedies or oriental medicine)^a^ - Preferred to seek Indigenous medicine care outside the clinic or health post - Consulted a traditional healer	Chile and Haiti	Use of traditional medicine indicated unmet health-care needs	(i) The possibility that traditional medicine was chosen by preference or because of a lack of access to medical care is not considered; and (ii) various practitioners whose therapies were subject to different levels of regulation and had different degrees of efficacy were combined into a single group

WHO: World Health Organization.

^a^ Techniques such as vibrational medicine, biomagnetism, reiki, iridology, Bach flower remedies and oriental medicine are classified as complementary medicines according to WHO terminology guidelines, which emphasize integration alongside conventional health care rather than its replacement.^6^^,37^

Frequently in surveys, traditional medicine was nested among the response options to a question on why the respondent did not seek or receive care from a conventional health-care provider (i.e. the reason for forgoing care). Other response options usually listed related to: (i) the availability of care (for example, the nearest clinic did not offer the services needed); (ii) geographical accessibility (for example, the clinic was too far away); (iii) affordability (that is, treatment was too expensive); and (iv) perceived fairness of, respect by and trust in services (for example, concerns about discrimination or mistreatment or a lack of trust). The proportion of the population who used traditional medicine when they last needed health care can be estimated from these responses. Furthermore, the proportion who mentioned the use of traditional medicine as a reason for forgoing conventional health care can be compared to the proportion who mentioned barriers to access, such as geographical accessibility and affordability. As a result, policy-makers can determine whether people are forgoing conventional health care because they prefer traditional medicine or experience barriers.

As currently constructed, however, most household surveys provide data on traditional medicine that are not directly comparable with each other or that are not practically useful beyond providing basic estimates of traditional medicine use. Although current household surveys often include information on traditional medicine, the formulation of the questions can limit the usefulness of the data. Table 1 provides examples of how traditional medicine was typically categorized in the surveys we reviewed, and highlights challenges in interpreting survey findings. The surveys typically presented traditional medicine as either a type of care received for an illness or accident or as a reason for not seeking conventional medical care. In some surveys, traditional medicine was recorded as meeting a health-care need but, as there were no follow-up questions on barriers to care, the presence of unmet needs may have been masked. Even when surveys included follow-up questions, there were variations in the questions used, which complicated the analysis of barriers to accessing health care. In addition, some surveys that incorporated follow-up questions on barriers to care classified traditional medicine use as an unmet need and may, therefore, have incorrectly implied that all traditional medicine use reflected gaps in access to conventional care rather than preferences. Consequently, the use of traditional medicine could be misinterpreted as either a substitute for conventional health care or an indicator of unmet needs, without it being considered as complementary. Table 1 also notes that surveys in Chile and Haiti, for example, grouped diverse traditional medicine practitioners into a single category, which made it difficult to distinguish between preference and access limitations as the reason for traditional medicine use, and to distinguish between types of traditional medicine with evidence on safety and effectiveness available and those without such evidence.

We identified several factors that limited the usefulness of survey data (Box 1), including: (i) a lack of specificity in descriptions of traditional medicine; (ii) limited understanding of the processes and outcomes of traditional medicine; (iii) a lack of clarity in identifying reasons for using traditional medicine; (iv) a lack of comparability between data from different surveys; and (v) the combination of different types of traditional medicine practitioner into a single group. These limitations make it difficult; (i) to understand the nuanced reasons why people choose traditional medicine; (ii) to determine whether traditional medicine is effective in meeting health needs; (iii) to inform policy decisions on the integration of traditional medicine into health systems; and (iv) to make comparisons between different studies and contexts. Here, we propose some minor modifications that could meaningfully improve the utility of existing survey questions.

Box 1 **Limitations of household survey data, review of population-based surveys reporting traditional medicine use, WHO Region of the Americas, 2010–2023**

***Lack of specificity***

Often survey questions did not specify the type of local traditional medicine provider consulted or product used (for example, spiritual healer or herbal medicine), which makes it difficult to determine the efficacy and level of regulation of the service or product.

***Poor understanding of the care process, its outcomes and patient satisfaction***

Typically surveys did not ask about the processes involved in traditional medicine or the outcomes of the care received from its providers. Consequently, it was difficult to know whether a prescription was given or care was effective, thereby making it impossible to determine if the person's health needs were met.

***Lack of clarity in identifying the reason for traditional medicine use***

When traditional medicine was given as a reason for forgoing conventional health care, the survey questions often failed to clarify whether this was due to the patient’s preference, barriers to accessing conventional health care, referral from a conventional health-care provider or a combination of these factors. This lack of clarity makes it hard for policy-makers to understand the true drivers of traditional medicine use.

***Lack of data comparability***

The data extracted from different household surveys were not directly comparable and were useful only for providing basic estimates of the level of traditional medicine use, which makes it difficult to conduct meaningful comparative studies.

***Practitioners of different types of traditional medicine combined into a single group***

Some survey questions combined practitioners whose therapies were subject to different levels of regulation and had different degrees of efficacy into a single group.

WHO: World Health Organization.

First, the survey questions should specify the type of locally relevant traditional medicine provider or product (for example, spiritual healer or medicinal plants) so that scientific data on the efficacy of the service or product can be assessed, and information on its local regulation and integration into health care can be determined (using information from other sources). If feasible, regardless of the type of provider, further questions should be asked about traditional medicine processes (for example, whether a prescription was given) and about the outcomes of the care received to determine whether care was effective and, thus, whether the individual’s health needs were met.

Second, when traditional medicine is included as a reason for forgoing conventional health care, questions should be asked to clarify whether traditional medicine was chosen because of the patient’s preference, barriers to conventional health care, referral by a conventional health-care provider, or a combination of these factors. This information would enable researchers to distinguish between these reasons and could inform policy-makers about the extent to which people use traditional medicine not by choice or referral but because of barriers to conventional health care.

Additional research on the typology of traditional medicine services and products, the quality controls applicable to traditional medicine and ways of measuring the integration of traditional medicine into health systems would be very useful and could help in modifying survey questionnaires, analysing data and devising policy. Until better data sources are available, making the best use of existing population-based surveys is a practical way to get a sense of who has access to, and is using, traditional medicine to meet their health needs and of the circumstances that lead to its ineffective use, thereby leaving people with unmet needs.

## Policy implications

Improvements in the collection and analysis of data on the use of traditional medicine can have an impact on health-care policy. In particular, better evidence can support the integration of effective traditional practices into the health system and help address unmet needs and gaps in care.

Our synthesis of current population-based data on traditional medicine use across the Americas highlights both patterns of use and persistent data gaps. However, the limited data available on the use of traditional medicine is further compromised by a lack of evidence on the benefits, efficacy and safety of traditional medicine modalities. This dual absence restricts the ability of policy-makers to make informed decisions about the integration and regulation of traditional medicine and the resources that should be allocated to it.

A key limitation of household surveys is that they probably underrepresent marginalized groups, such as ethnic minorities, migrants and people in informal settlements, because of barriers such as language and population mobility. This underrepresentation could lead to underestimates of traditional medicine use and less accurate data, which may result in policies that overlook underserved communities. These problems should be tackled by improving the inclusivity of surveys and by using complementary data sources.

Improvements in the collection of data on traditional medicine, including reasons for seeking traditional medicine and its outcomes, can help policy-makers tailor strategies for its integration into health care. For example, high usage of traditional medicine in places where integration policies exist may point to good access to treatment, whereas high demand in settings where there is little formal support for traditional medicine may highlight the need for further system integration.^38^ In both situations, understanding what is driving traditional medicine use can guide its safe, effective and culturally sensitive integration into health care.

Information about the prevalence of traditional medicine use and about the specific types being used can lead to the better allocation of resources. Moreover, a data-driven approach could guide the implementation of a balanced strategy that addresses both the demand for traditional medicine and gaps in conventional health care. For instance, if a particular type of traditional medicine is used widely and is effective, countries could invest in its regulation and in training to ensure it is safe and provides high-quality health care. Simultaneously, resources could be directed to address gaps in health-care provision where traditional medicine is being used because of barriers to conventional health care. For example, for long waiting times, a lack of trust or cultural preferences.

Understanding the circumstances in which people choose traditional medicine can help ensure it is used safely and effectively and does no harm. Survey questions could be modified to help determine whether traditional medicine use is driven by patient preferences, barriers to conventional health care or referrals. The availability of these data, combined with the administrative records of facilities or hospitals and community needs assessment reports, could help health care become more holistic, culturally responsive and people-centred. In addition, it is important to acknowledge the diverse reasons individuals seek different forms of care, such as cultural factors, affordability and perceived effectiveness.^1^

As people often switch between conventional and traditional medicine, particularly in low- and middle-income countries, there is a vital need for better coordination and communication between different health-care sectors to ensure the continuity and effectiveness of care. Moreover, the availability of robust data on traditional medicine use can be helpful in efforts to educate both health-care providers and the public; health workers could learn about the benefits and limitations of traditional medicine, and the public could be informed about safe and effective practices.

## Conclusions

By addressing the limitations of population-based survey questions on traditional medicine and extracting the maximum amount of information possible on its utilization from existing surveys, policy-makers can develop strategies to satisfy unmet health-care needs, reduce inequity and foster safe, integrated health systems. The result would be not only better health-care delivery but also the preservation of traditional knowledge as a valuable public health resource.
